# Importance of evaluating protein glycosylation in pluripotent stem cell-derived cardiomyocytes for research and clinical applications

**DOI:** 10.1007/s00424-021-02554-x

**Published:** 2021-04-08

**Authors:** Maia I. Kelly, Mustafa Albahrani, Chase Castro, Ellen Poon, Bin Yan, Jack Littrell, Matthew Waas, Kenneth R. Boheler, Rebekah L. Gundry

**Affiliations:** 1 CardiOmics Program, Center for Heart and Vascular Research, Division of Cardiovascular Medicine, Department of Cellular and Integrative Physiology, University of Nebraska Medical Center, Omaha, NE 68198 USA; 2 Department of Medicine and Therapeutics, Centre for Cardiovascular Genomics and Medicine, Hong Kong Hub of Pediatric Excellence, Lui Che Woo Institute of Innovative Medicine, The Chinese University of Hong Kong, HKSAR Hong Kong, China; 3 Department of Computer Sciences, The University of Hong Kong, Hong Kong, HKSAR China; 4 The Second Affiliated Hospital and Yuying Children’ s Hospital of Wenzhou Medical University, Wenzhou, Zhejiang China; 5 Present Address: Princess Margaret Cancer Centre, University Health Network, Toronto, ON M5G 0A3 Canada; 6 Whiting School of Engineering, Department of Biomedical Engineering, Traylor Building, The Johns Hopkins University, Baltimore, MD 21205 USA

**Keywords:** Stem cell-derived cardiomyocytes, Protein glycosylation, Mass spectrometry

## Abstract

Proper protein glycosylation is critical to normal cardiomyocyte physiology. Aberrant glycosylation can alter protein localization, structure, drug interactions, and cellular function. The in vitro differentiation of human pluripotent stem cells into cardiomyocytes (hPSC-CM) has become increasingly important to the study of protein function and to the fields of cardiac disease modeling, drug testing, drug discovery, and regenerative medicine. Here, we offer our *perspective* on the importance of protein glycosylation in hPSC-CM. Protein glycosylation is dynamic in hPSC-CM, but the timing and extent of glycosylation are still poorly defined. We provide new data highlighting how observed changes in hPSC-CM glycosylation may be caused by underlying differences in the protein or transcript abundance of enzymes involved in building and trimming the glycan structures or glycoprotein gene products. We also provide evidence that alternative splicing results in altered sites of glycosylation within the protein sequence. Our findings suggest the need to precisely define protein glycosylation events that may have a critical impact on the function and maturation state of hPSC-CM. Finally, we provide an overview of analytical strategies available for studying protein glycosylation and identify opportunities for the development of new bioinformatic approaches to integrate diverse protein glycosylation data types. We predict that these tools will promote the accurate assessment of protein glycosylation in future studies of hPSC-CM that will ultimately be of significant experimental and clinical benefit.

**Supplementary Information:**

The online version contains supplementary material available at 10.1007/s00424-021-02554-x.

## Overview of protein glycosylation

Protein glycosylation occurs in all eukaryotic cells [103]. The presence of a glycan moiety at the appropriate site within a glycoprotein is critical for proper protein folding and stability. Glycoproteins play critical roles in cell adhesion, signaling, and immune responses [2, 82, 103, 123]. Cell–cell adhesion, self/non-self-recognition, molecular trafficking and clearance, evasion of host immune recognition, and receptor activation are examples of glycan-mediated events [63, 103].

*N-*linked and *O-*linked are two major types of protein glycosylation. The process of *N-*linked glycosylation occurs co-translationally as proteins are translocated through the endoplasmic reticulum (ER) (reviewed in [46, 89]). An *N-*linked precursor glycan is transferred from a dolichol-phosphate onto asparagine residues within the consensus sequence [(Asp (N)-x-Ser (S)/Thr (T)/Cys (C)), where x can be any amino acid except proline] within the polypetide chain [9, 62]. A Val (V) in the third position has also been proposed as a consensus sequence for glycosylation [125], but our bioinformatic analysis indicates that both the NxC and NxV motifs occur at a lower rate than expected in predicted surface/extracellular proteins and at a higher rate than expected in predicted intracellular proteins. This pattern is opposite to what is observed for the NxS/T motif and suggests that the presence of NxC/V sequence motif alone offers no evidence of surface protein *N-*glycosylation [105]. After transfer of the glycan onto the protein, glycoproteins traverse the ER and Golgi apparatus, and the oligosaccharide is trimmed by glycosidases in a highly coordinated fashion. The glycans are then modified with sialic acid residues in the Golgi. In contrast, *O-*linked glycans are normally added post-translationally on Ser (S) and Thr (T) residues to proteins within the Golgi, although some types of *O-*glycosylation are initiated in the ER [21]. There is no known general consensus sequence for *O-*linked glycosylation, although a specific consensus-sequence in the epidermal growth factor protein domains has been described [21, 34].

**Fig. 1:**
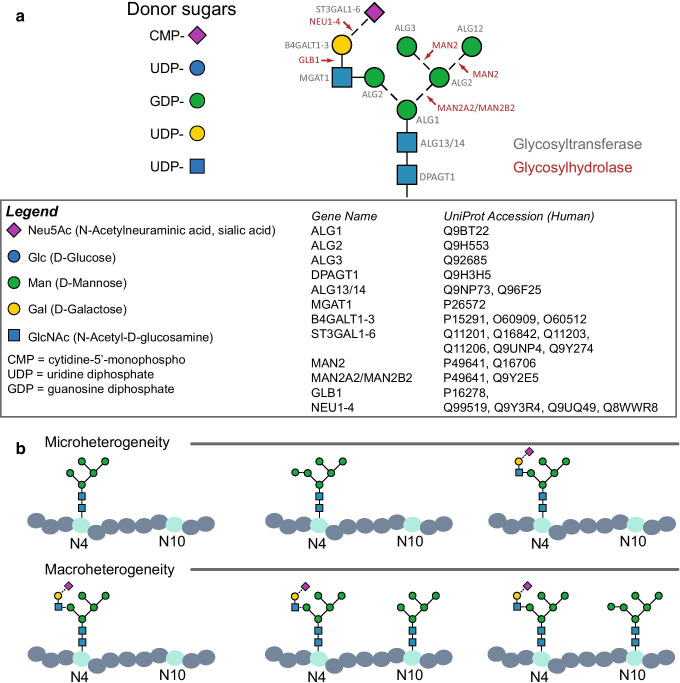
Overview of protein *N-*glycosylation. **A** Glycan structures are generated by complex interactions among enzymes (shown in red and grey) that build and trim the structures using a pool of donor sugars. An example *N-*glycan is shown here with the corresponding donor sugars and enzymes that would be required to generate or trim the structure. **B**. Examples of glycan microheterogeneity and macroheterogenity in glycoproteins are provided

Unlike RNA and protein, glycan synthesis is not a template-driven process. Rather, the resulting glycan structure is defined by the actions of available glycan-modifying enzymes (there are > 300 human glycosyltransferases and glycosidases), which are influenced by a variety of factors. These include the availability of nucleotide sugar donors used for glycan biosynthesis, and the spatial organization and structure of the secretory pathway, both of which can be affected by cellular stressors (reviewed in [90]) (Fig. 1A). This complex regulation within the glycan biosynthetic pathway results in tremendous diversity in glycan structures and glycoprotein proteoforms (i.e., different forms of proteins generated by genome sequence variations, splicing, and post-translational modifications that include glycosylation). Different glycan structures can occupy a single site on a protein (i.e., microheterogeneity). There can be varying levels of site occupancy and different combinations of glycan structures across the protein sequence (i.e., macroheterogeneity) (Fig. 1B). Beyond their canonical intracellular location within the ER-Golgi apparatus, some glycosyltransferases are secreted and function as extracellular enzymes in the circulation [48], further confounding the observed heterogeneity.

## Protein glycosylation in cardiomyocyte development and disease

Most cell surface and secreted proteins are predicted to be glycosylated and can contain both *N-* and *O-*glycans [103, 105]. In the heart, ion channels at the cell surface are required for propagation of action potentials and subsequent contraction of the myocardium [102]. Differential sialylation (i.e., the covalent addition of sialic acid to the terminal end of a glycan) may modulate cardiac voltage-gated sodium channel activity throughout development or between cardiac chambers. Treatment with neuraminidase to remove sialic acids from rat neonatal atria and adult atria and ventricles results in depolarized potentials akin to those measured for channels from neonatal ventricles [95]. Treatment of rat neonatal cardiac myocytes with neuraminidase to remove sialic acids also causes altered cellular calcium concentrations and contractile function [121]. Distinct glycan profiles have been reported for rat neonatal and adult ventricles [17]. Rat left ventricle and atria express distinct β_1_-adrenergic receptor proteoforms, a receptor whose cleavage—and downstream signaling—is partially mediated by *O-*glycosylation [80]. Finally, DNA microarray analysis has shown that glycosylation-associated genes (glycogenes) are highly regulated in cardiomyocytes and are modulated uniquely between newborn and adult cardiac tissue types [72].

Cardiac protein sequence mutations can lead to disruptions of *N-*glycosylation sites in ion channel proteins and impair their function and localization at the cell surface. Perturbation of *N-*glycosylation sites of potassium/sodium hyperpolarization-activated cyclic nucleotide-gated (HCN) channel 2 leads to loss of its expression at the cell surface in HEK293 cells [49, 74]. Mutations in *N*-glycosite motifs in three different ion channels—voltage-dependent calcium channel subunit alpha-2/delta-1 (CACNA2D1), voltage-dependent sodium channel type 5 subunit alpha (SCN5A), and potassium channel subfamily K member 2 (KCNK2)—lead to decreased steady-state cell surface densities of these proteins and loss of catalytic activity [8, 18, 96, 114]. Glycosylation site mutations in potassium voltage-gated channel subfamily E member 1 (KCNE1) give rise to a form of the Long-QT syndrome, a heart rhythm disorder [91]. Human induced pluripotent stem cell (hiPSC) lines derived from patients with Long-QT syndrome recapitulate the electrophysiological characteristics of this disease phenotype, and hiPSC-CMs showed altered glycosylation and trafficking of the potassium voltage-gated channel subfamily H, member 2 (KCNH2, HERG) [65, 68]. Separately, the KCNE2 protein associates with and modulates potassium channels. The absence of glycosylation at site N6 of KCNE2, due to a variant in the third position of the *N-*glycosite sequon, is primarily responsible for increased susceptibility of KCNH2 to the antibiotic sulfamethoxazole [79, 92]. Based on these findings, glycosylation of KCNE2 is proposed to protect the HERG channel from high-affinity block of sulfamethoxazole [79].

Beyond mutations in cardiac ion channels, defects in the glycosylation pathway that attaches glycans to proteins or lipids have been reported (reviewed in [28]). Collectively referred to as congenital disorders of glycosylation (CDG), these defects encompass a variety of inborn metabolic disorders. Type I CDGs are related to defects in the assembly or transfer of the dolichol lipid-linked glycan to either proteins or lipids. Type II CDGs are aberrations in trimming and processing of protein-bound glycans in the ER or Golgi. Approximately 20% of CDG have been associated with cardiac complications (reviewed in [61]), including defects in glycosylation enzymes that give rise to dilated cardiomyopathy, hypertrophic cardiomyopathy, and endocardial sclerosis [19, 39, 59, 71, 76, 100].

Altogether, these studies reveal how proper protein glycosylation is critical to normal cardiomyocyte function. Changes in glycosylation status of proteins affect cardiomyocyte physiology by altering their localization, function, and drug interactions. Therefore, the study of protein glycosylation is essential if we are to truly understand the biological mechanisms that affect protein function.

## Human pluripotent stem cell-derived cardiomyocytes and protein glycosylation

Human pluripotent stem cell-derived cardiomyocytes (hPSC-CM) can be produced in nearly unlimited quantities for the study of cardiomyocyte protein function, early cardiomyocyte development, cardiac disease, and toxicology testing [12, 14, 16, 27, 64, 73, 75, 101]. The use of hiPSC-CM enables personalized modeling of cardiovascular disease, drug response, and, potentially, regenerative medicine strategies. The utility of hPSC-CM for each of these applications is, however, inextricably tied to how accurately they model the correct cell type (e.g., progenitor, cardiomyocyte) from the appropriate anatomical region of the heart (e.g., pacemaker, left or right atria, left or right ventricle, septum, apex), the relative developmental stage (e.g., fetal, neonatal, adult), and how well the cells recapitulate the expected phenotype (e.g., disease, response to drug).

Several recent comprehensive reviews of glycosylation in undifferentiated hPSC underscore the importance of glycosylation in pluripotency and early differentiation and the value of using hiPSC models to understand tissue-specific mechanisms in CDG [11, 30, 31, 77, 111]. Despite mounting evidence for its relevance and importance, analysis of protein glycosylation in hPSC-CMs remains limited (Table 1).

**Table 1:** Summary of published studies that have evaluated glycans or protein glycosylation in pluripotent stem cell-derived cardiomyocytes. Days refer to the number of days of differentiation at which point the samples were analyzed for glycans/glyco proteins. For analytical methods, MALDI matrix-assisted laser desorption/ionization; PGC-LC porous graphitized carbon liquid chromatography; RP-LC reversed phase liquid chromatography

PMID	Model	Day(s)	Genetic Background	Analytical Method	Data Type
*Glycans*					
28,374,933 [1]	Mouse ESC-CM	0	CMP-Sia synthetase-/- and ±	Laser-induced fluorescence detection	Released glycoprotein glycan structures
25,488,666 [85]	Human iPSC-CM	42	Pompe disease vs Normal	MALDI mass spectrometry	Released glycoprotein glycan structures
31,058,489 [88]	Human iPSC-CM	10	Normal	Laser-induced fluorescence detection	Released glycosphingolipid glycan structures
26,378,261 [41]	Human iPSC-CM	18	Normal	2D HPLC and MALDI mass spectrometry	Released glycoprotein glycan structures
25,357,199 [42]	Mouse iPSC-CM	16	Normal	2D HPLC and MALDI mass spectrometry	Released glycoprotein glycan structures
28,509,371 [45]	Human iPSC-CM	0,7,15	Normal	Laser-induced fluorescence detection	Released glycoprotein glycan structures
31,972,267 [6]	Human iPSC-CM	20–100	Normal	PGC-LC mass spectrometry	Released glycoprotein glycan structures
*Glycoproteins*					
28,509,371 [45]	Human iPSC-CM	0, 7,15	Normal	RP-LC mass spectrometry	Cell surface glycoproteins, (detection of non-glycosylated peptides)
32,157,205 [83]	Human ESC-CM	15–90	Normal	RP-LC mass spectrometry	Cell surface glycoproteins, deglycosylated peptides
31,972,267 [6]	Human ESC, iPSC-CM;	Cardiac organoid (day not stated)	Normal	RP-LC mass spectrometry	Re-analysis of glycopeptide data from PMID: 28,916,735 [70] which performed proteomic analysis

Currently, only seven studies have evaluated glycosylation in hPSC-CM by released glycan analysis. This strategy is useful for determining glycan composition or isomeric structure but does not identify the protein(s) to which the glycans were attached. To determine the role of sialylation in early development, *N-*glycans from a mouse embryonic stem cell (mESC) line lacking the CMP-Sia synthetase (Cmas; Cytidine 5′-monophosphate (CMP)-sialic acid synthetase, an essential enzyme involved in the biosynthesis of glycans containing sialic acids) were evaluated at one time point by high-performance multiplexed capillary gel electrophoresis with laser-induced fluorescence (xCGE-LIF) [1]. Loss of Cmas resulted in undifferentiated mESC with an increase in oligoLacNAc-capped glycans and glycans with terminal galactosyl sugars. However, loss of Cmas did not inhibit the cardiomyocyte differentiation of these cells. This enzyme thus appears dispensable in early murine cardiomyocyte development. In another disease-based study, *N-*glycans from hPSC-CM generated from patients with Pompe disease displayed a reduced diversity of multiantennary structures compared to control hPSC-CM [85]. The diseased hPSC-CM also contained an abundance of the trimannose complex glycan precursors, which were undetectable in control hPSC-CM. These data suggest that Pompe hPSC-CM have the *N-*linked glycan core, but do not undergo further branching and extension as in the control hPSC-CM. These data provide evidence of Golgi-based glycosylation defect in Pompe, providing a potential link between Pompe cardiomyopathy and cardiomyopathies observed in CDG.

Three studies completed global glycome characterization of undifferentiated hPSC compared to hPSC-CM at a single timepoint of differentiation. A study of glycosphingolipid glycans in hiPSC and hiPSC-CMs using xCGE-LIF revealed that monosialodihexosylganglioside (GM3), the b-series ganglioside GD3, sialyl neolactotetraosylceramide (Lc4), and nLC4 were highly abundant in hiPSC-CM compared to hiPSC [88]. Two comparative studies used MALDI to analyze *N-*glycans in human and mouse iPSCs compared to hPSC-CM. In mouse, Kawamura et al. described changes in *N-*glycans that occurred during miPSC-CM differentiation including a decrease in high mannose and exposed GlcNAc glycans and an increase in exposed galactose and sialylated glycans. In human, an increase of α2,3-sialylation in hiPSC-CM compared to hiPSC was identified which was correlated with the observed higher expression of ST3Gal3 in the hiPSC-CM comparatively [41, 42]. Further, increase in terminal fucose was observed more frequently in hiPSCs than hiPSC-CMs. Altogether, these studies suggest that protein glycosylation changes as pluripotent stem cells differentiate to cardiomyocytes.

Analysis of glycans at multiple timepoints of hPSC-CM differentiation has provided greater insights into glycome dynamics during in vitro differentiation and maturation. Using xCGE‐LIF, Konze et al. examined early myogenic and cardiomyogenic commitment by comparing glycans from hPSC-CM at differentiation days 0, 7, and 15 and from primary human cardiomyocytes [45]. They reported 62 N*-*glycan structures and observed three structures unique to hPSC compared to hPSC-CM, including β1,3-linked galactose, α2,6-linked sialic acid, and complex fucosylation. By day 15 of differentiation, hPSC-CMs had an increase in α2,3-sialylation and bisecting GlcNAc residues when comparing cells from days 0 and 7. Our group used porous graphitized carbon liquid chromatography electrospray tandem mass spectrometry (PGC-LC–ESI–MS/MS) to separate and identify glycan isomers to develop a broader view of glycome dynamics in committed cardiomyocytes [6]. We identified 265 N*-* and *O-*glycan structures from primary human cardiomyocytes and from hPSC-CM at differentiation days 20–100. Of the structures analyzed, 23% of the glycans were shared among hPSC-CM and primary cardiomyocytes, while different sialylation linkages were observed between the two sample types. During hPSC-CM differentiation, few significant differences were observed between high-mannose and hybrid classes of glycans; however, glycans within the complex mono-antennary class structures significantly decreased over time. Glycans within the complex bi-, tri- and tetra-antennary classes included those that increased and others that decreased over time. This included structures with α2,8-linked sialylation motif that increased 100-fold over time of differentiation.

**Fig. 2:**
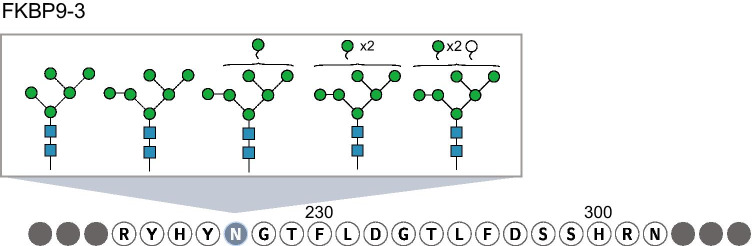
Microheterogeneity at glycosylation sites complicates the identification of glycosylation moieties. As an example, one glycopeptide of FKBP9-3 found in 3D cardiac organoids has multiple sites of glycosylation, including one site that has five different glycans

Three studies have generated proteomic data suitable for analysis of *glycoproteins* from hPSC-CM (see Table 1). Mills et al. performed a proteomic analysis of 3D cardiac organoids and human heart tissue [70]. In our re-analysis of their data, we used Byonic to search against a glycan structure library constructed by amending the standard glycan library with structures identified in our glycomics analysis of hPSC-CM and human heart cells and tissue [6]. Novel patterns of glycoprotein microheterogeneity emerged from this analysis. Examples included HSPG2, FINC, and LG3BP glycopeptides that differed between 3D organoids and primary heart tissue [7]. Here, we report another example of microheterogeneity that emerged from this analysis. Specifically, FKBP9-3 has five different glycan compositions present at site N227 in the 3D cardiac organoids (Fig. 2). The physiological relevance of this microheterogeneity is yet unknown, but this finding is presented to emphasize the concept that protein glycosylation status is complex and cannot be predicted from transcriptomic or proteomic datasets alone.

Second, in the study by Konze et al. described above for released glycans*,* the authors also performed an enrichment of cell surface sialylated glycoproteins followed by tryptic digestion to identify sialo-glycoproteins from hiPSC and from hPSC-CM at days 7 and 15 of differentiation. This approach, which did not analyze glycopeptides, but instead examined the non-modified peptide fraction, identified 879 proteins. Among these were proteins that increased (e.g., HCN4, AGRN, HSPG2) and decreased (e.g., TENM4) in abundance at day 15 compared to day 7.

**Fig. 3:**
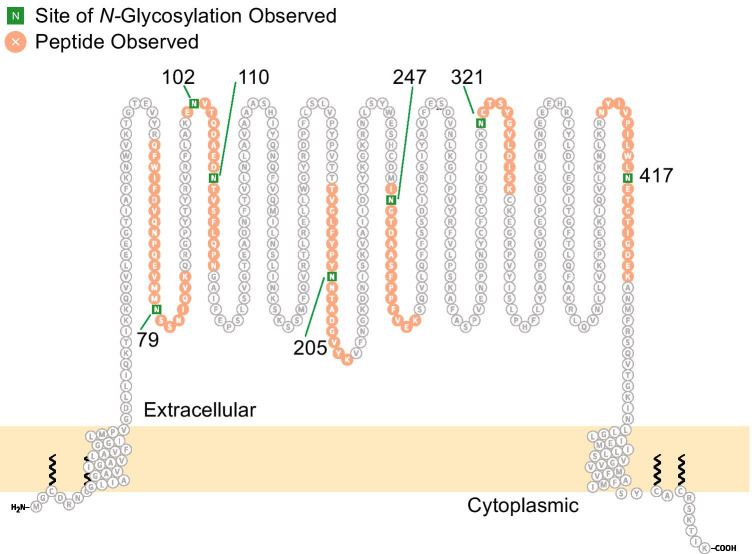
Schematic representation of cell surface *N-*glycopeptides and *N-*glycosites in CD36 identified by CSC analysis of hPSC-CM. Image generated with Protter [78]

Finally, we have identified > 650 cell surface *N-*glycoproteins on hPSC-CM collected from days 20–100 of differentiation (published and unpublished) using the cell surface capture (CSC) technology [29, 115]. The CSC approach enriches for extracellular *N-*glycopeptides and can be used to identify sites of glycosylation, but not the glycan moiety. Among published data, we report that CD36 represents a marker of matured, mitochondria-rich hPSC-CM that is largely absent from undifferentiated hPSC and early hPSC-CM [83]. The extracellular domain of CD36 is predicted to contain 10 N*-*glycosylation sites (UniProt), including some that are critical for trafficking to the surface membrane [37] and biological function [56]. In our CSC analyses of hPSC-CMs, we identify seven *N-*glycosylation sites in total for CD36 (Fig. 3). Sites Asn-247, Asn-321, and Asn-417, which have been shown to be important for membrane trafficking [37], were only observed in day 31 + hPSC-CM. Future studies that implement methods for the analysis of intact glycopeptides will be required to confirm the glycan compositions present at each of these sites. Collectively, the complementary released glycan and glycopeptide approaches provide evidence that protein abundance and glycosylation are dynamic during the differentiation and maturation of hPSC-CM.

## Expanding the assessment of protein glycosylation in hPSC-CM

### Strategies to assess protein glycosylation

Routine assessments of hPSC-CM combine analyses at the transcript, protein, and metabolite level with phenotypic and physiological recordings [10, 27, 40, 58, 81, 107]. However, neither glycan structures in general, nor the presence of a specific glycan structure at a specific glycosite within a protein, can be directly predicted from transcriptomic, proteomic, or metabolomic studies. Multiple analytical approaches for the identification, characterization, and quantification of protein glycosylation, therefore, have been developed. These approaches can be broadly characterized according to the level of structural detail that can be achieved and whether the amino acid residue occupied by the glycan can be determined.

Lectins (proteins that recognize and bind glycans) can be used for high-throughput screening (lectin arrays) and imaging (reviewed in [32]). However, lectins recognize glycan motifs, do not provide information about complete monosaccharide composition, and are limited to targets for which reagents are available. Currently, no *N-*glycan structure-specific lectins and few *O-*glycan structure-specific lectins are available [16, 33, 39, 52, 68].

To inform the specific enzymes in the glycan biosynthetic pathway needed to generate the structures in hPSC-CM, a full structural characterization is necessary. This level of detail is typically achieved when glycans are released and empirically measured either by high-performance liquid chromatography (HPLC) or mass spectrometry (MS), which do not require anti-protein or anti-glycan affinity reagents. While these analytical techniques are apt for glycan characterization, structural elucidation, and quantitation, the methods differ by whether they can determine glycan composition (identity and quantity of monosaccharides within the glycan) or full structural characterization (composition and linkage and branching points of monosaccharides).

Detection of fluorescent labeled released glycans in conjunction with HPLC can be used to profile glycans and, when coupled with exoglycosidase treatment, can identify new glycan structures [23, 45, 60]. MS-based approaches include the analysis of deglycosylated peptides, intact glycopeptides, released glycan compositions, and released glycan structures. Each of these approaches require distinct analytical platforms that have been extensively reviewed elsewhere (reviewed in [7, 13, 52]). MS methods that enable determination of glycan structure include chemical derivatization of specific residues such as linkage specific sialic acid derivatization [20], all monosaccharide permethylation [26], or reduction of free glycans [38], followed by separation using hydrophilic interaction (HILIC) or PGC liquid chromatography prior to MS [38, 104].

Methods for the analysis of deglycosylated peptides from hPSC-CM include the cell surface capture (CSC) technology, periodate oxidation, and aniline‐catalyzed oxime ligation, and related approaches, which specifically enrich for *N-*glycoproteins localized at the cell surface [84, 115, 124, 124]. Numerous alternative strategies enrich glycopeptides from whole cell or tissue lysate, but such approaches do not provide evidence for subcellular localization akin to that provided by the cell surface glycoprotein focused methods [55, 66, 108, 110, 113, 118, 120]. Methods for intact glycopeptide analysis by MS enable determination of monosaccharide compositions present at specific sites on a protein, but do not typically generate sufficient information to fully characterize glycan structures or structural isomers [36, 43, 44, 50, 86, 94, 97].

**Fig. 4:**
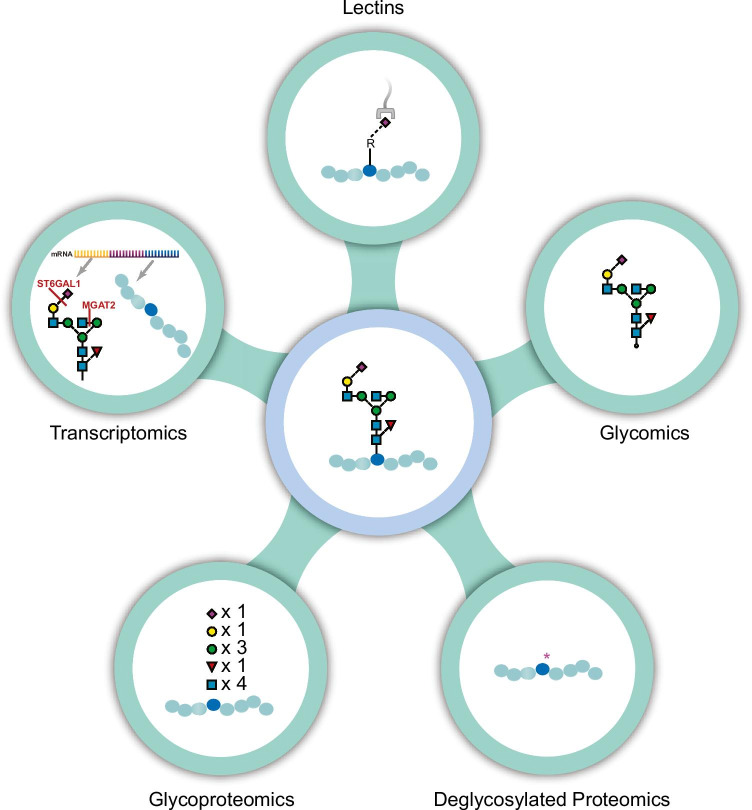
Protein glycosylation can be studied by various approaches, which altogether provide complementary views of the glycoproteome. Lectins can recognize certain motifs or epitopes on the glycans. Glycomics methods include fluorescent detection of labeled glycans, and MS analysis of labeled or unlabeled glycans that can give structural, composition, or isomer specific information. Cell surface capture and other methods involving enrichment and deglycosylation of glycoproteins allow for glycosylation site localization and protein identification. Glycoproteomics analyses of intact glycopeptides can identify glycan compositions present at specific sites within the protein. Transcriptomics can inform regulation of glycoproteins and enzymes within the biosynthetic pathway of glycosylation

Currently, no singular strategy is available that enables the simultaneous identification of peptide sequence and full glycan structure characterization of glycoproteins [51, 60]. Rather, multiple complementary strategies are required to fully elucidate the glycan structures present on specific sites within a protein (Fig. 4). For these reasons, there has been limited integration of complementary approaches for the characterization of the hPSC-CM glycome. The remainder of this perspective focuses on the exploration of new data and integration of disparate data types to provide further evidence for the relevance of protein glycosylation when evaluating hPSC-CM for research and clinical applications.

### Exploring dynamic protein glycosylation throughout hPSC-CM differentiation

As described above, we and others determined that *N-*glycans are dynamic throughout differentiation of hPSC-CM [6, 41]. However, a limitation of these studies is that the glycans were released from the protein backbone prior to MS analysis, making it impossible to determine to which proteins the glycans are attached. To complement these data and provide new insights regarding whether the glycoproteins present on the cell surface change during differentiation, we combined data from two complementary approaches, CSC technology and RNA-seq.

**Fig. 5:**
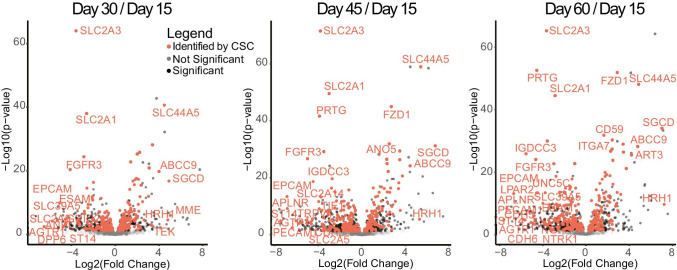
Cell surface *N-*glycoproteins are dynamic over stages of differentiation. Volcano plots of RNA-seq data for 2057 transcripts with SPC scores of 3, 4. Of those, the cell surface *N-*glycoproteins that were identified by CSC are highlighted in orange. Plots show log_2_ fold change versus the -log_10_ *p*-values for hPSC-CM differentiation days 30, 45, or 60 compared to day 15. Transcripts for which the transcript levels are significantly changed (*p* < 0.05) by log_2_ fold change > 4 or a log_2_ fold change <  − 4 are labeled with gene symbols

Application of the CSC technology to hPSC-CM collected from 18 experiments on 9 timepoints between days 10 and 93 of differentiation resulted in the identification of 627 cell surface *N*-glycoproteins identified in at least two experiments (methods described in [83]). RNA-seq analyses were performed on hPSC-CM collected from four timepoints of differentiation, days 15, 30, 45, and 60. Data from both approaches were integrated by annotating each protein identified by CSC with the transcript level from the RNA-seq experiments. To visualize whether transcripts for predicted cell surface proteins are quantitatively changing, genes for transcripts from the RNA-seq analysis were annotated with their surface prediction consensus (SPC) score [106]. SPC scores range from 0 to 4 with scores of 3 or 4 representing a high confidence prediction in surface localization. SPC score of 0 indicates a protein either has a predicted localization other than the cell surface or is a GPI-anchored or extracellular matrix protein that does not contain transmembrane domains. Volcano plots were generated featuring all transcripts with SPC scores of 3 or 4 (Fig. 5). These data demonstrate the transcriptional levels of many predicted cell surface transmembrane proteins are dynamic throughout differentiation.

The CSC technology provides experimental evidence that a protein is localized to the cell surface. However, it is possible that differential detection of a peptide (i.e., apparent difference in abundance) by this method could be due to changes in protein localization or in the glycan structure, either of which can interfere with the glycopeptide capture or release. RNA-seq data do not provide direct measurement of protein abundance or localization but rather reveal mRNA levels or alternative splicing of mRNA. The annotation of transcripts from RNA-seq data with the SPC score does not provide experimental evidence that the proteins for these transcripts are localized at the cell surface in these cells. Instead, it serves as a filter for predicted cell surface proteins. Moreover, mRNA and protein abundance at the cell surface are not always correlated as cell surface proteins can be sequestered (e.g., CD36) and further processed after translation in ways that would alter their detection by CSC [56].

**Fig. 6:**
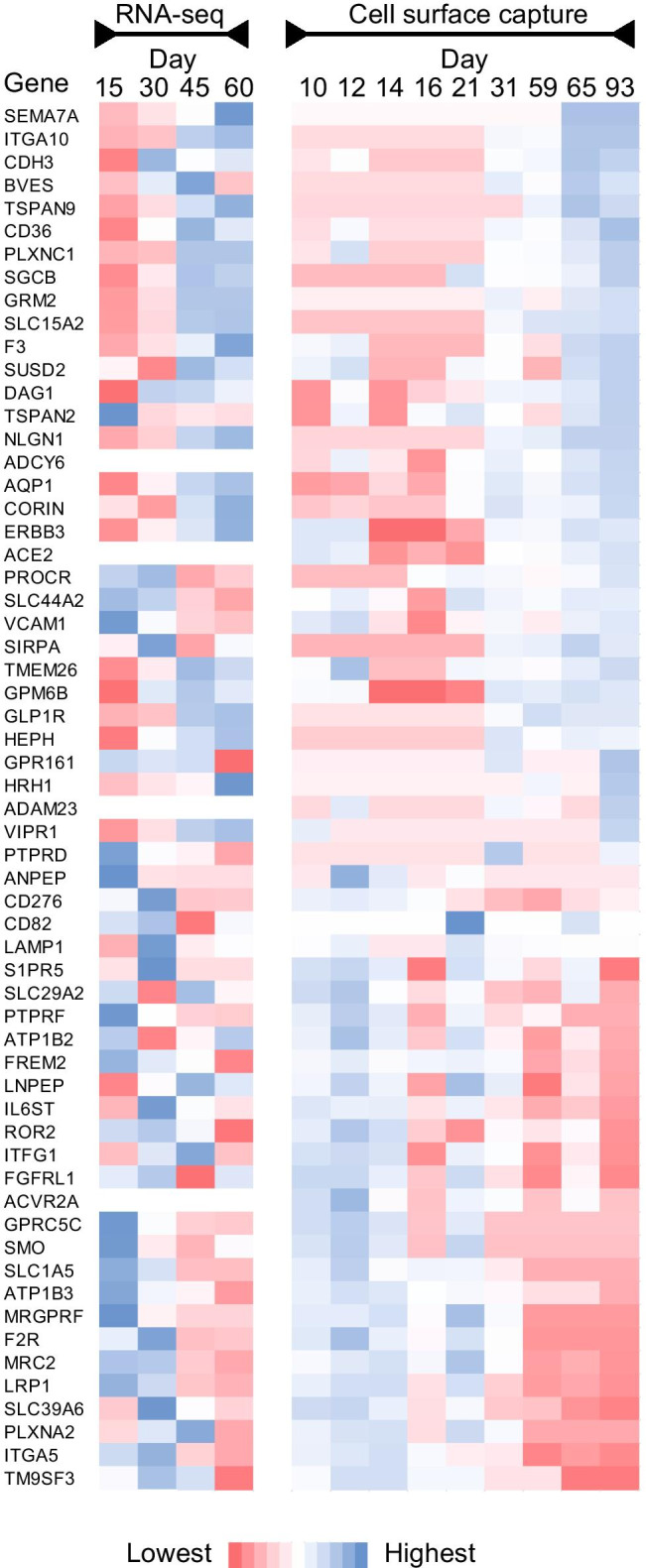
Relative transcript and glycopeptide abundance for selected cell surface proteins during hPSC-CM differentiation. Count data from RNA-seq and peptide spectrum matches from CSC were averaged across technical replicates and normalized to maximum detection for each protein by each technique

With these caveats in mind, further examination of RNA-seq data for cell surface *N-*glycoproteins identified by CSC reveals consistencies and discrepancies in the trends among stages of differentiation (Fig. 6). Besides CD36, many proteins found by CSC with a trend of increasing abundance during differentiation displayed a similar trend by RNA-seq. These include ITGA10, TSPAN9, GRM2, SCL15A2, NLGN1, and GLP1R which were only detected in day 30 + hPSC-CM by CSC and showed increased mRNA abundance with differentiation time. Similarly, SEMA7A was detected in only day 60 + hPSC-CM and showed low but increasing mRNA levels by RNA-seq. Proteins detected at days 10–21 of differentiation by CSC include GPRC5C and SMO for which RNA-seq also shows a decreasing trend over time. Intriguing are ADCY6, ACE2, ADAM23, and ACVR2A which were detected by CSC but had mRNA levels below the threshold for consideration in these studies. The reason for this discrepancy is unknown, particularly since ADCY6 transcripts were found to increase with time of differentiation when evaluated independently by qPCR (online resource 1). Despite this incongruity, these data provide evidence for why protein-level assessments are important even in the age of next-generation sequencing technologies. Finally, LAMP1 and ERBB3 are examples where mRNA expression levels were modest (counts ranged 1272–1849 and 258–1829, respectively) at all timepoints, yet were detected by very few spectra (≤ 10) in CSC at days 59–93. The cause for this inconsistency is unknown but could possibly be related to alterations in *N-*glycosylation composition or site occupancy as discussed below.

### Glycosylation pathway enzymes during hPSC-CM differentiation

**Fig. 7:**
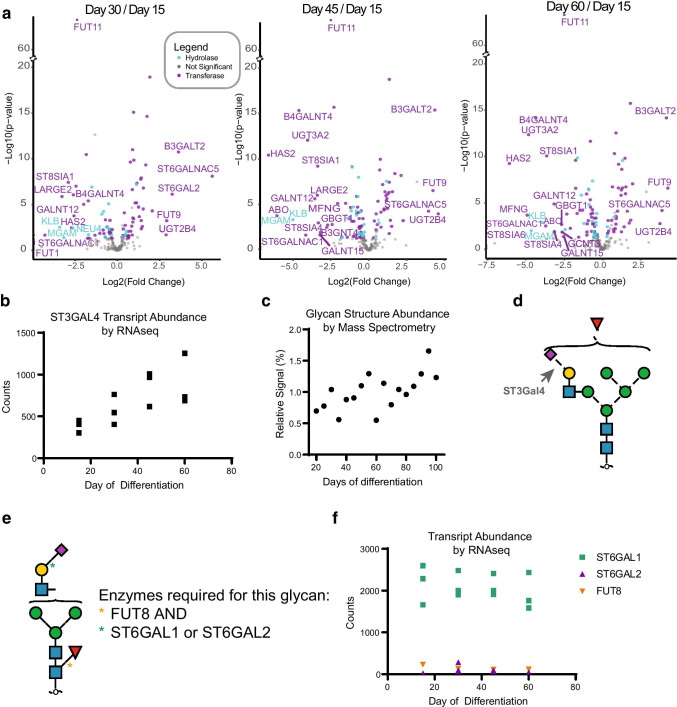
Glycosylation pathway enzymes are dynamic over stages of hPSC-CM differentiation. **A** Volcano plots of RNA-seq data for 237 transcripts annotated as either glycosylhydrolases or glycosyltransferases. Plots show log_2_ fold change versus the -log_10_ *p*-values for hPSC-CM differentiation days 30, 45, or 60 compared to day 15. Transcripts for which the transcript levels are significantly changed (*p* < 0.05) by log_2_ fold change > 4 or a log_2_ fold change <  − 4 are labeled with gene symbols. **B** Transcript abundance for ST3GAL4 from RNA-Seq data showing an increase with time of differentiation. **C** Relative abundance of the glycan structure shown in (**D**) across days of hPSC-CM differentiation. **E** Glycan structure we previously identified [6] in primary cardiomyocytes but not hPSC-CM for which the combination of FUT8 and either enzyme ST6GAL1 or ST6GAL2 would be required to generate the linkages indicated by (*). **F** RNA-seq data from hPSC-CM showing that only ST6GAL1 is robustly expressed in hPSC-CM. The minimal expression for ST6GAL2 and FUT8 in hPSC-CM is consistent with the lack of detection of the glycan shown in panel e in these cells

The mRNA and protein levels of enzymes associated with the glycosylation pathway are dynamically regulated during hPSC-CM differentiation. To inform whether our reported changes in glycans during hPSC-CM differentiation [6] are potentially due to changes in glycosylation machinery, we assessed the RNA-seq data for transcripts of glycosylation enzymes. Volcano plots were generated featuring human glycosidases or glycosyltransferases (annotated according to Carbohydrate-Active enZYmes Database [53]; Fig. 7A). Overall, of the 237 transcripts identified in the RNA-seq data that play a role in the glycan biosynthetic pathway, the abundance of 106 transcripts significantly changes during the time course of differentiation.

Several observations emerge from the integration of these RNA-seq data with our structure-based glycomics analysis. First, our RNA-seq data reveal that the glycosylation enzyme beta-galactoside alpha-2,3-sialyltransferase 4 (*ST3GAL4*) increases with differentiation time (Fig. 7B). This result is consistent with our previously reported glycan structure data, where relative signal abundance for glycan structures that contain α2,3-sialylation (linkages which are synthesized by ST3GAL4) increases over time of differentiation (example of one structure shown in Fig. 7C, D). While we do not know to which proteins this glycan structure is attached, the importance of this enzyme for cardiomyocyte function is evidenced in a previous finding that targeted deletion of *St3gal4* in mice leads to decreased sialylation on voltage-gated sodium channels that alters channel gating and is consistent with increased susceptibility to arrhythmia [25].

Second, we previously observed differences in glycan structures between hPSC-CM and primary isolated human cardiomyocytes that we thought might be related to differences in expression of glycosylation enzymes required to generate these glycan structures. Specifically, we proposed that a combination of the fucosyltransferase FUT8, expressed in tandem with ST6GAL1 or ST6GAL2, would be required to generate a structure found uniquely in the cardiomyocytes isolated from human hearts. The structure requiring both enzymatic reactions is shown in Fig. 7E. Our new RNA-seq data further refines this hypothesis, as only *ST6GAL1* is robustly expressed in hPSC-CM (Fig. 7F). Therefore, due to the minimal expression of transcripts for *ST6GAL2* and *FUT8* in hPSC-CM, the glycan found uniquely in primary isolated human cardiomyocytes (Fig. 7E) is likely synthesized primarily by these two enzymes.

While we do not yet know the physiological significance of the glycosylation changes observed for hPSC-CM over time of differentiation, nor the differences between hPSC-CM and primary cardiomyocytes, the analyses presented here provide examples of how the integration of different data types can further refine hypotheses for future studies that integrate changes in the pathway of glycosylation (transcriptomics, proteomics) with measurements of the end-products (glycoproteomics, glycomics).

### New approaches are needed to promote assessment of protein glycosylation

Analysis of protein glycosylation is challenging and not yet routinely used to assess hPSC-CM. As described above, protein glycosylation is the result of a complex biosynthetic pathway, and no single analytical approach currently enables the determination of full structural detail of the glycan attached to specific amino acids within a protein. Rather, complementary approaches that separately determine released glycan structure and glycan composition for a specific site must be integrated to fully determine glycan structures present at a specific glycosite on a protein.

Analytical approaches that provide structural detail are necessary to inform which metabolic precursors and enzymes within the glycosylation pathway are required to generate glycan structures. But these methods do not directly provide insight into whether the glycosylation enzymes are regulated transcriptionally or if there is a change in the pool of nucleotide sugars used for glycan biosynthesis. Combined transcriptomic, metabolomic, and proteomics approaches are required to inform whether differences in glycoproteins observed among cell types or conditions are due to transcriptional regulation of enzymes within the biosynthetic pathway or of the glycoprotein itself, or availability of metabolic precursors.

**Fig. 8:**
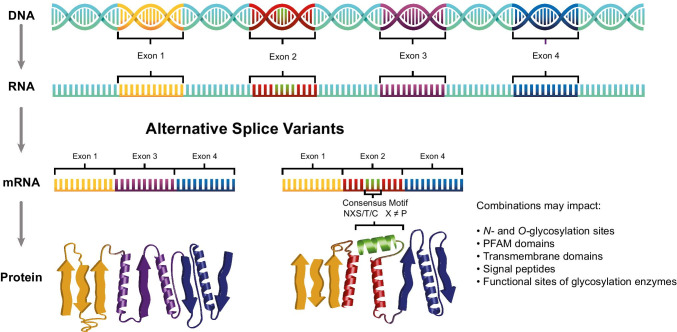
Graphical overview of alternative splicing and examples of effects that rearrangements can have on sequence and features especially relevant for glycoproteins

RNA-seq data can inform splicing events and, consequently, sample-specific databases for glycoproteomics studies. Alternative RNA splicing, where intron and exon elements are rearranged and joined to alter the mRNA coding sequence, results in the synthesis of multiple protein sequences from a single gene (Fig. 8). Alternative splicing can alter the presence or location of glycosite motifs, signal peptides, or transmembrane domains within the protein, each of which are critical for glycoprotein localization and function. Compared to proteins synthesized from canonical mRNA sequences (i.e., non-spliced mRNA), proteins translated from alternatively spliced mRNAs contain different amino acid sequences and may have different biological functions [99]. Alternative splicing is a key mechanism that diversifies protein function during cardiomyocyte development. During post-natal development of mouse heart, genes involved in membrane organization and vesicular trafficking are regulated by alternative splicing [33]. Alternative splicing events in adult and fetal human heart are linked to protein synthesis and cell-cycle regulation [109].

**Fig. 9:**
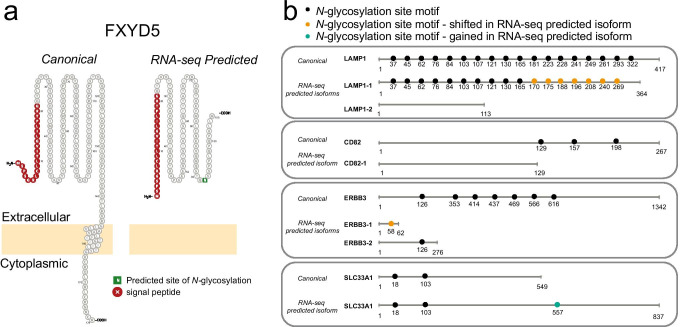
Examples of alternative splice isoforms from the RNA-seq analysis of hPSC-CM to illustrate implications of alternative splicing on protein glycosylation. **A** FXYD5 canonical sequence is predicted to contain a single transmembrane domain and no sites of *N-*glycosylation. The RNA-seq predicted isoform contains no transmembrane domain and a single site of *N-*glycosylation. Images generated using Protter [78]. **B** The isoforms for LAMP1, CD82, ERBB3, and SLC33A1 predicted by RNA-seq contain altered sequences due to splice junction events that lead to predicted differences in the presence of *N-*glycosylation motifs

To predict whether alternative splicing may affect sites of glycosylation in hPSC-CM proteins, we created a library of protein isoform sequences informed by RNA-seq data using a pipeline of STAR, rMATS, and JCAST [22, 47, 93]. Our analysis reveals that 19,068 predicted sites of *N-*glycosylation (considering only NxS and NxT sequon motifs) across 3951 proteins are putatively different between the alternatively spliced isoforms compared to the canonical sequences (online resource 2). We found 2,913 (~ 35%) *N-*glycosylation sites shifted in position in the predicted isoforms, while 5126 sites (~ 62%) among 1764 proteins were lost, and 198 sites (~ 2%) among 164 proteins were gained. Of those proteins whose sites were lost or gained, 122 are proteins with an SPC score of 3–4, meaning they are high confidence predicted surface proteins. As an example, the canonical sequence of FXYD domain-containing ion transport regulator 5 (FXYD5) contains one transmembrane domain and no predicted *N-*glycosites (UniProt). RNA-seq data predict an alternative isoform that contains a single *N-*glycosite motif in the extracellular domain, and by Phobius [30], it is predicted to be entirely extracellular without a transmembrane domain (Fig. 9A). Previous studies have shown this protein is *O-*glycosylated [98], but to our knowledge, *N-*glycosylation and lack of transmembrane domain for this protein have not been reported. As shown in Fig. 9B, RNA-seq predicts isoforms of LAMP1, CD82, ERBB3, and SLC33A1 each contain lost and/or gained sites of *N-*glycosylation in comparison to their canonical isoforms. The loss of sites for LAMP1 and ERBB3 could explain why the CSC approach detects very few *N-*glycopeptides for these proteins, while the mRNA levels are robust, as described above.

**Fig. 10:**
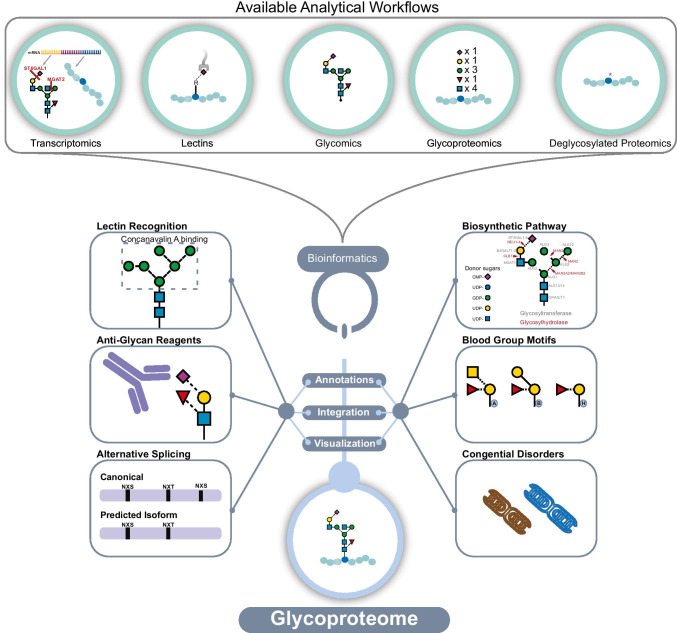
Overview of the types of data integrations that are necessary to obtain a comprehensive view of protein glycosylation. Various analytical workflows are used to generate complementary data. New tools are needed to annotate, integrate, and visualize results from RNA-seq, glycomics, and glycoproteomics with contextual information related to blood group antigens, congenital disorders, lectin recognition motifs, anti-glycan reagents, biosynthetic pathway steps, and the impact of alternative splicing on the resulting glycan structures present on glycoproteins

While the functional relevance of these putatively lost or gained glycosite motifs is unknown, nor have they been confirmed at the protein level, these data highlight the need for better tools to facilitate these types of analyses. Currently, the interrogation of RNA-seq data for predicted glycosite changes and integration of those predictions with experimental data requires technical knowledge in different data analysis pipelines. Glycome data warehouses, data visualization, and data analysis tools are becoming increasingly available and richly populated [3–5, 15, 35, 57, 69, 112, 117, 119, 122]), but we still lack efficient ways to fully integrate all of the data types illustrated in Fig. 10. Specifically, tools that facilitate the use of sample-specific RNA-seq are needed to inform possible changes to protein sequence and glycosylation site occupancy that could be experimentally verified by MS or other approaches. Data analysis tools that make it easy to determine the presence of blood group motifs from glycomics data could impact the evaluation of hPSC-CM for regenerative medicine. Finally, annotation of experimental data with information related to available lectin or other anti-glycan reagents would facilitate orthogonal approaches to visualize the location of specific glycan motifs within complex tissues and organs. Altogether, the development and implementation of bioinformatics tools that facilitate the analysis and integration of data from disparate analytical workflows should facilitate the routine evaluation of protein glycosylation in hPSC-CM and other biological samples.

## Conclusions and future prospects

The biological importance of glycosylation during development and disease is an emerging field of investigation with implications for hPSC-CM. In this perspective, we present evidence that glycosylation is dynamic during in vitro differentiation and maturation of hPSC-CM. However, our view of protein glycosylation within hPSC-CM remains limited as does our understanding of the implications of aberrant glycosylation in hPSC-CM models as compared to primary CM. Considering the biological relevance of glycosylation for cardiomyocyte function, studies of hPSC-CM are expected to benefit from the inclusion of strategies to assess protein glycosylation. Appropriate strategies would include transcriptomics to assess mRNA levels of proteins and enzymes involved in the biosynthesis of glycosylation as well as glycoproteins, MS to determine glycan structure and glycopeptide identity, and lectin and other anti-glycan affinity reagents to inform spatial localization. Continued studies to define protein glycosylation, its regulation, and physiological implications will be important for advancing the utility of hPSC-CM for research and clinical applications. We expect new facile approaches for integrating the vast and disparate datasets required to generate an accurate view of sample-specific protein glycosylation will promote the broad implementation of studies of protein glycosylation for hPSC-CM. As these approaches evolve and become more readily accessible, we advocate that *protein glycosylation should be considered when evaluating the suitability and applications of hPSC-CM products for drug testing, modeling human development and disease, or regenerative medicine.*

## Supplementary Information

Below is the link to the electronic supplementary material.

Online Resource 1 Supplemental methods and figure (DOCX 30.9 KB)

Online Resource 2 Table of RNA-seq predicted isoforms for hPSC-CM (XLSX 8660 KB)

## Data Availability

Data are provided in the online resources.
