# Supplementary material for: Importance of evaluating protein glycosylation in pluripotent stem cell-derived cardiomyocytes for research and clinical applications
Source: Pflugers Arch. 2021 Apr 8;473(7):1041–59. doi: 10.1007/s00424-021-02554-x (PMC8245383; doi:10.1007/s00424-021-02554-x)
Supplement: Supplementary file 1 — Online Resource 1 Supplemental methods and figure (DOCX 30.9 KB) [file 424_2021_2554_MOESM1_ESM.docx]

**Online Resource 1**

**Supplemental Methods and Materials**

*hPSC-CM differentiation*

Undifferentiated human embryonic stem cells (hESCs, Line H7) were maintained on Matrigel (BD Biosciences, Sparks, MD)-coated surfaces at 37°C and 5% CO2 in E8 medium plus supplements (Thermo Fisher Scientific, Waltham, MA). In vitro differentiation into cardiomyocytes was performed as previously described [2]. Prior to the initiation of differentiation, a total of 70,000 cells were plated onto individual wells of a 6-well plate in E8 medium. Four days later, E8 medium was changed to RPMI/B27 medium lacking insulin. CHIR99021 (6 μM, final; Selleckchem, Houston, TX) was added to the cells from days 0 to 2, followed by cultivation in RPMI/B27 medium lacking insulin for 24 hours. On days 3-5, IWR-1 (10 μM; Selleckchem) was added to the medium before switching back to RPMI/B27 lacking insulin. On day 8, cardiomyocyte (CM) cultures were maintained in RPMI/B27 medium containing insulin (Thermo Fisher Scientific), which was changed every 3-4 days.

*RNAseq Analysis of hPSC-CM*

RNA-seq analyses (n=3 for each time point) were performed as previously described [2], using poly-A^+^ RNA extracted from CMs at days 15, 30, 45 and 60 of in vitro differentiation. In brief, total RNA was extracted using an RNeasy micro kit (Qiagen, Hilden, Germany) and polyA^+^ RNA isolated. cDNA libraries were prepared using the KAPA Stranded mRNA-Seq Kit according to the manufacturer’s protocol. Adaptor-ligated libraries were enriched by 15 cycles of polymerase chain reaction (PCR), denatured and diluted to an optimal concentration. Samples were then applied to the HiSeq PE Cluster Kit v4 with cbot for cluster generation on the flow cell. Illumina HiSeq SBS Kit v4 was used for Pair-End 101 bp sequencing performed at the HKU Genome Centre. The raw RNA-seq data were aligned to the human genome using TopHat. The read counts at the gene level were defined by GENCODE annotation and extracted using the HTSeq tool. Genes identified by RNA-seq and the corresponding gene names (according to standard nomenclature) were used for analyses with SurfaceGenie [3]. Only transcripts with a counts per million (CPM) mapped value >10 and a threshold for expressed genes as determined automatically in DESeq1 were considered in further analyses.

A library of protein isoform sequences informed by RNA-seq data using a pipeline of STAR, rMATS, and JCAST [21, 46, 90]. Transcripts were aligned to the genome using STAR. Alternative splicing events were then found using rMATS by comparing samples at each stage of development. Finally, JCAST was used to reverse translate the alternatively spliced sequences and select the ones most likely to be found based on frameshifts and the presence of start and stop codons.

*Glycopeptide identification*

Using data from previous glycomics studies [1], a glycan library was constructed with both glycans found in the study and standard Byonic glycan libraries. Raw files were obtained from PXD005736. Byonic was used to search for glycopeptides with the Uniprot Human database with added decoys, one missed trypsin cleavage, fully specific trypsin cleavage and HCD fragmentation. Tolerances were set at 20 ppm precursor mass tolerance and 20 ppm fragment mass tolerance. The modifications used were fixed carbamidomethyl (C) and variable

**Online Figure 1. qPCR of ADCY6 for days 0-30 of hPSC-CM differentiation.** RNAs were extracted from three biological replicates of each day point. Then cDNAs were synthesized. Real-time PCR was performed on each cDNA in duplicate. Mean Ct value was obtained for each cDNA, then used for 2^-ΔΔCt^. Average fold change for each day point was calculated, as well as standard deviation in Excel. *GAPDH* and RNA from H7 undirected differentiated (UDD) cells were used as reference gene and control sample, respectively.
